# Comparative clinical data for gingivitis treatment using gels from *Ocimum sanctum* (Tulsi) and chlorhexidine (CHX)

**DOI:** 10.6026/973206300171091

**Published:** 2021-12-31

**Authors:** BA Deepika, Jaiganesh Ramamurthy, Nadathur Duraisamy Jayakumar, S Rajesh Kumar

**Affiliations:** 1 Saveetha Dental College and Hospitals, Saveetha Institute of Medical and Technical Sciences, Saveetha University, Chennai, Tamilnadu, India

**Keywords:** *Ocimum sanctum*, dental plaque, gingivitis, chlorhexidine, local delivery, Tulsi

## Abstract

*Ocimum sanctum* (Tulsi) has various properties like anti bacterial, anti inflammatory, anti oxidant for curing diseases. It is a plant with known medicinal value in Indian system of medicine. Therefore, it is of interest to evaluate the effectiveness of *Ocimum sanctum* with Chlorhexidine (CHX) which is a standard material for the treatment of gingivitis. We used 30 gingivitis subjects divided into 2 groups. Group I used Tulsi gel (n= 15) and Group II used CHX gel (n = 15) for treatment. Tulsi and CHX gel use was advised for 1 month. The Clinical parameters assessed were gingival Index (GI), plaque Index (PI), probing depth (PD) and clinical attachment loss (CAL) assessed at a time interval of 30 days. Statistical analysis was completed using the SPSS software 23.0. Data showed that GI and PD for Tulsi and CHX in pre and post groups are not significant with p > 0.05. Moreover, PI is not significant with p>0.05 among pre Tulsi, pre CHX and post CHX. However, data is significant with p<0.05 for Tulsi group. CAL is significant with p<0.05 among pre/post Tulsi groups. However, this is not significant with p>0.05 among pre/post CHX groups. Data shows that 2% of Tulsi is effective in reducing gingival bleeding and inflammation. Thus, clinical data shows that Tulsi gel is promising for the treatment of gingivitis.

## Background:

Plaque is the main factor for gingivitis (gingival inflammation). Oral hygiene failure leads to plaque formation [1]. The mechanical supra gingival plaque control measures includes toothbrush, floss, wood sticks, and interdental brushes. Oral hygiene maintenance is in plaque control measures [2]. There is increasing inclination towards biofilm properties and microbial colonies [3]. Different stages of dental biofilm formation are (a) absorption of bacterial molecules to host tooth surface [4-6]; (b) transport of bacteria to tooth surface [7-9]; (c) adherence of late colonies to early colonies [10-12]; (d) multiplication of the attached organisms [13-18] and (e) active detachment [19,20]. Gingivitis is the inflammation of gingiva without apical migration of junction epithelium [21]. Prolonged use of Chlorhexidine leads to brown colour stains on the teeth and change in pattern of taste. It also leads to formation of calculus and swelling of the parotid gland [22]. Plant extracts are used as the main component in mouthwash to reduce gingival inflammation and gel forms are mostly used to reduce periodontitis. These products have low side effects because of their herbal nature [23]. Some Indian plant species that contain antimicrobial activities are as follows they are Aloe vera, *Piper betel*, *Piper nigrum*, *Syzygium aromaticum*, *Coriandrum sativum*, *Eucalyptus globules*, *Allium sativum*, *Curcuma longa*, *Camellia sinensis*, *Alliumcepa*, *Carica papaya*, *Solanum tuberosum* & *Ocimum sanctum* [24]. *Ocimum sanctum* (Tulsi) belongs to the basil family Lamiaceae. Tulsi is an aromatic shrub [25]. Tulsi has both medicinal properties and spiritual properties [26]. The 2 types of Tulsi are Sri Tulsi and Krishna Tulsi. Sri Tulsi has green coloured leaves and Krishna Tulsi has purple coloured leaves [27]. Tulsi is available in various forms like dried leaves powder; fresh leaves and herbal tea [28]. Tulsi was used as a mouthwash to control plaque and gingival conditions [29]. A 4% of prepared Tulsi plant extract showed highest antibacterial activity against *Streptococcus mutans* [30]. Thus, Tulsi is considered as "The master of the herbs" for its magical healing properties and anti-inflammatory properties [31 - check with author]. Therefore, it is of interest to document the comparative clinical data for gingivitis treatment using gels from *Ocimum sanctum* (Tulsi) and chlorhexidine.

## Materials & Methods:

### Preparation of 2% Ocimum sanctum gel:

Data for the preparation is shown in Table 1 (see PDF).

### Preparation of supercritical fluid:

**Figure 1:**
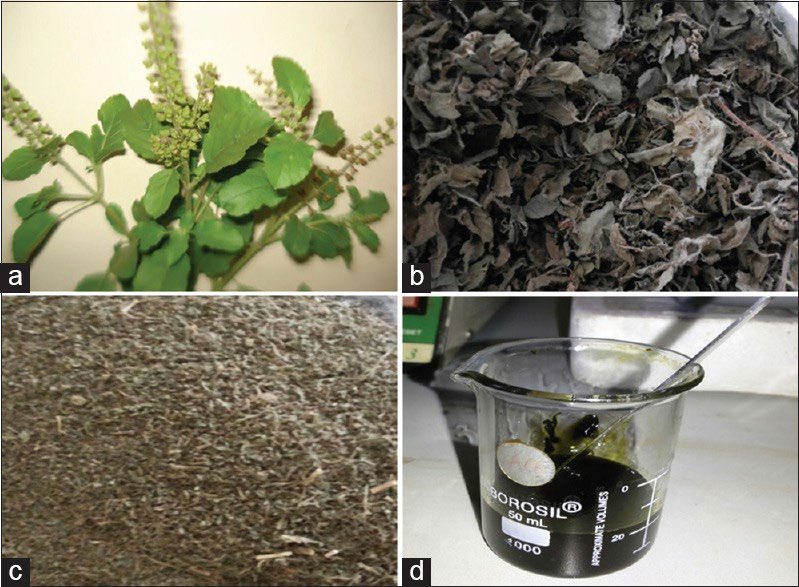
The image shows (a) Tulsi leaves; (b) dried Tulsi leaves; (c) powdered tulsi and (d) Tulsi gel

**Figure 2:**
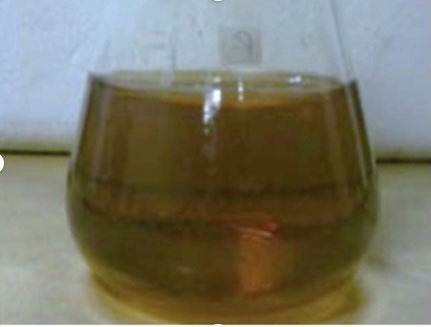
The image depicts the preparation of Supercritical fluid - 250 g of tulsi powder taken and soaked in 1000 ml of ethyl alcohol for 48 hours.

**Figure 3:**
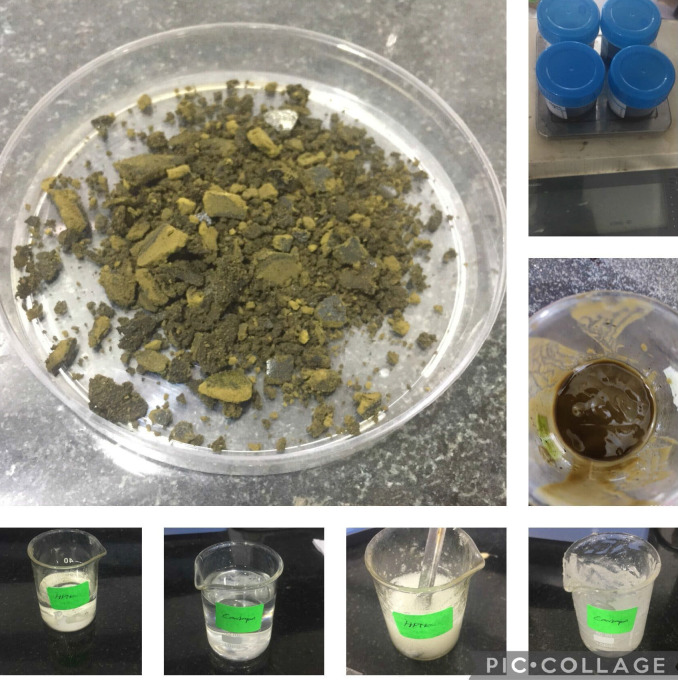
The image shows the gel prepared using Tulsi leaf dried extract, carbopol, HPMC, prepared Tulasi gel is stored in Air tight container boxes and stored in the fridge.

250 g of Tulsi powder is taken and soaked in 1000 mL of Ethyl alcohol for 48 hours. Filter with Whartman's filter. Filter liquid evaporated - Supercritical Fluid and stored in the fridge is shown in Figure 1 and Figure 2. Preparation of Ocimum sanctum gel: Carbopol 940 soaks it in distilled water containing 0.2% sodium benzoate throughout the night. HPMC solution was added. Propylene glycol was added to it. 2ml of SCF (Homogenized) was added. Tri ethanol amine was added in drops. Check for pH 6-6.5.The gel was stored at ambient temperature. The prepared gel is firm for the time interval of six months. Alteration in pH changes were noted and adjusted according to the protocol (Figure 3).

### Assessment of physical properties of 2% Ocimum sanctum gel:

The formulations were assessed for different evaluation tests. They evaluate the physical appearance, homogenous nature, spreadability, grittiness, ability to extrude, and pH evaluation. Estimation: The Physical examinations are Hue (color) and Aspect (look) was assessed. Lubricity: Lubricity is estimated by a wooden block apparatus. The apparatus has a pulley at one of its ends. Basic methodology is to slide and draw the properties of *Ocimum sanctum* (Tulsi) gel. Two grams of the prepared gel was taken and kept on the bottom slide. The *Ocimum sanctum* (Tulsi) gel was fitted into the bottom slide; another slide with the same measurements was taken and a hook was attached to it. One kilogram weighing scale was kept on the top of 2 slides for a period of 5 minutes. Hence air bubbles were eliminated to produce a constant form of gel film among slides. Excess gel was removed from the edges. The top plate was put through a jerk of 80 grams with the help of a string which is joined with a hook. The time period taken to envelope a distance of 7.5 centimeters by the top plate was noted in seconds. A shorter interval means better spreadability nature.

Spreadability was evaluated using the approved formula: S = M x L/T Where, S = Spreadability, M = Weight applied the pan (tied to the upper portion of slide), L = to what length moved by the glass slide and T = Time in seconds taken to separate the slides from each other.

### Homogeneity:

Once the formulation is set in a container Homogeneity was examined by visible monitoring. It was evaluated for visible and tangible aggregates. It is categorized as good, fair, poor. For good it triple plus scale, fair means double plus, poor means single plus.

### Extrudability:

The formulation was stored in a clean aluminium collapsible one ounce tube and it has a nasal tip of 5 mm opening slot. It was assessed by calculating the quantity of the gel expelled through nib when applied with a load of one kilogram was kept. The expelled gel was collected in a container and weighed for the measurement. The expelled gel was measured in percentages, and categories were assigned.

### Determination of viscosity:

Viscosity of the formulation was evaluated at 25°C using Brookfield digital viscometer. The dimensions were taken in the established speed settings. The settings range from 10 to 100 rotations per minute. A time interval of 30 seconds among 2 consecutive speeds in a declining manner is used.

### pH Measurement:

2.5 grams of the *Ocimum sanctum* (Tulsi) gel was taken and measured exactly and then poured into 25 ml of water. It was stored for 2 hours. The pH was measured using a standard pH meter.

### Study design:

The study design includes a parallelized controlled clinical trial for the subjects from outpatient department of periodontics, Saveetha dental college and hospitals for the eligibility criteria for the study population are as follows:

### Inclusion criteria:

1. Patients with generalized chronic gingivitis.

2. Patients in the age group of 20-65 years.

3. Systemically healthy subjects with Gingival index score, Plaque index score and sulcular bleeding index score > 1 at the time of examination.

### Exclusion criteria:

1. Patients with periodontitis.

2. Smokers.

3. Antibiotic therapy within last 6 months of the study

4. Pregnant and lactating women.

5. Patients undergone or having undergone periodontal therapy within the last 6 months of study.

With the above inclusion criteria and exclusion criteria a total of 30 subjects were included in the study. The subjects were asked to massage the gingiva with Tulasi gel and CHX gel 2 times a day for 1 month.

### Study group:

Group I:Tulasi gel

Group II: CHX gel

The assessment criteria included Gingival Index (GI) score, Plaque Index (PI) score, Probing Depth (PD) and Attachment Loss (AL) score that were measured as pre and post *Osmium sanctum* (Tulasi) gel and Chlorhexidine (CHX) gel.

### Statistical analysis:

Differences between the study groups were statistically analyzed by SPSS Software 23.0 version; Paired t-test was used to analyse the difference between the groups. Mean and Standard Deviation were assessed for statistical analysis and the results are tabulated.

## Results and Discussion:

Plant extracts are possible sources of novel antimicrobial components especially against bacterial microorganisms. An important feature of plant extracts and their constituents is hydrophobicity, which makes them divide the lipids portion of the cell membrane of bacteria and mitochondria, interrupting the structures of cells and making them more absorbent. Plants have different forms of bioactive compounds. It has different forms of phytochemical compounds [32]. It is of interest to assess the efficacy of *Ocimum Sanctum* (Tulsi) Gel compared to Chlorhexidine (CHX) Gel for the management of gingival disease patients. *Ocimum Sanctum* (Tulsi) gel is effective, demonstrating its potential use as efficient, and in addition used as standard control for the management of periodontitis. A Triple-blinded Randomized control trial to assess the effectiveness of *Ocimum Sanctum* (Tulsi) and Chlorhexidine (CHX) mouthwash used 4% w/v *Ocimum Sanctum* (Tulsi) and 0.12% Chlorhexidine (CHX) mouthwash. They show that *Ocimum Sanctum* (Tulsi) mouthwash was efficient in decreasing gingival condition and also decreasing the plaque levels as Chlorhexidine (CHX) mouthwash [33]. Ipsita *et al.* showed that the antimicrobial activity against *A. actinomycetemcomitans* & *P. gingivalis* at 8% concentration of *Ocimum Sanctum* (Tulsi) plant extract. Thus, *Ocimum Sanctum* (Tulsi) is an adjunct to mechanical therapy in the interruption and management of periodontal conditions [33].

Ahirwar *et al.* showed the effectiveness of *Ocimum Sanctum* (Tulsi) as a root canal medicament for primary molar teeth and evaluated it with respect to triple antibiotic paste. *Ocimum sanctum* (Tulsi) is known to have better results due to their antimicrobial properties and anti-inflammatory properties. Hence, it is used as a root canal medicament in primary teeth [34]. Mallikarjun *et al.* evaluated the antimicrobial efficacy of *Ocimum Sanctum* (Tulsi) with doxycycline as standard against periodontal microorganisms like *Aggregatibacter Actinomycetemcomitans*, *Prevotell aintermedia*, and *Porphyromonas gingivalis*. *Ocimum Sanctum* (Tulsi) at 5% and 10% concentrations showed better inhibition zones against *Aggregatibacter Actinomycetemcomitans*. They showed smaller inhibition zones against *Prevotella intermedia*, and *Porphyromonas gingivalis*. Hence, *Ocimum Sanctum* (Tulsi) is utilized as an efficient adjunct and in addition to the regular periodontal treatment [35]. Kamyab *et al.* discussed the anti inflammatory properties of *Ocimum sanctum* (Tulsi). It also discussed hepato protective properties and Gastric properties of *Ocimum Sanctum* (Tulsi). *Ocimum Sanctum* (Tulsi) is a medicinal plant with different forms of applications in the olden period as medicines. Nowadays, it is proved to be efficient against Diabetes mellitus, cancer, hepatic injury, bronchitis, gastric ulcer and also in hypertension [36]. Kalita *et al.* conducted a study to assess the antimicrobial properties of Tulsi, Pochotia and Neem against oral pathogens. The Plant extract was obtained from the hot continuous extraction method. The apparatus used is "Soxhlet". The oral microbes used were *E. faecalis, P. fluorescens, A. boumani, S. paucimobilis, K. kristinae, K. Oxytoca & B. subtilis*. Oral microbes were collected and poured in Mueller Hinton plates. The Plant extract were then poured into wells. Ciproflaxcin and Dimethyl sulfoxide were used as +ve and –ve controls. He concluded that all plant extracts used showed antibacterial effect against oral microbes [37].

Singh *et al.* discussed the uses of *Ocimum sanctum* seeds. Oil was extracted from seeds. Oil has anti histaminic activity and anti inflammatory activity because of dual suppression of arachidonate metabolism. The oil has antipyretic activity due to the inhibition of prostaglandin. It also has antiulcer activity. The oil has proven to be efficient against formaldehyde and in addition to induced arthritis. The oil also has immuno modulatory, hypotensive and anticoagulant properties [38]. Ramamurthy *et al.* showed *Ocimum Sanctum* (Tulsi) gel have possible anti-oxidant and anti-inflammatory effects. It is less toxic than brine shrimp nauplii. *Ocimum Sanctum* (Tulsi) proved to be the most favourable agent for the therapy of periodontal conditions [39].

*Ocimum Sanctum* (Tulsi) is equally effective against pathogenic gram +ve and gram -ve bacteria. Use of Tulsi in oral and systemic diseases, anti diabetic, wound recuperating movement, radio-defensive impact, cancer prevention agent, antimicrobial, gastroprotective, inflammatory, eye issue, renal issue, mental issue, skin issue, Tulsi use in oral health tooth ache, periodontal issue, anticariogenic impact, candidiasis, oral submucous fibrosis and ulcer. Therefore, it is of interest to evaluate the effectiveness of *Ocimum sanctum* with Chlorhexidine (CHX) which is a standard material for the treatment of gingivitis. The Clinical parameters assessed were gingival Index (GI), plaque Index (PI), probing depth (PD) and clinical attachment loss (CAL) assessed at a time interval of 30 days. Statistical analysis was completed using the SPSS software 23.0. Data showed that GI and PD for Tulsi and CHX in pre and post groups are not significant with p > 0.05. Moreover, PI is not significant with p>0.05 among pre Tulsi, pre CHX and post CHX. However, data is significant with p<0.05 for Tulsi group. CAL is significant with p<0.05 among pre/post Tulsi groups. However, this is not significant with p>0.05 among pre/post CHX groups. Data shows that 2% of Tulsi is effective in reducing gingival bleeding and inflammation. Thus, clinical data shows that Tulsi gel is promising for the treatment of gingivitis.

## Conclusion:

We report that 2% of Tulsi is effective in reducing gingival bleeding and inflammation. Thus, clinical data shows that Tulsi gel is promising for the treatment of gingivitis.

## References

[1] Loe H (1965). The Journal of Periodontology.

[2] Parwani SR (2013). Journal of Indian Society of Periodontology.

[3] Nadell CD (2016). Nat Rev Microbiol.

[4] Al-Hashimi I (1989). Archives of Oral Biology.

[5] Li J (2003). Oral Microbiology and Immunology.

[6] Vacca LK (2001). Caries Res.

[7] Busscher HJ (1997). Advances in Dental Research.

[8] Jenkinson HF (1997). Critical Reviews in Oral Biology & Medicine.

[9] Davey ME (2000). Microbiol Mol Biol Rev.

[10] Guggenheim M (2001). Appl Environ Microbiol.

[11] Socransky SS (1998). J Clin Periodontol.

[12] Bradshaw DJ (1998). Infection and Immunity.

[13] Allison DG (2003). The biofilm matrix Biofouling.

[14] Scheie AA (1994). Adv Dent Res.

[16] Beighton D (1986). Archives of oral biology.

[17] Bradshaw DJ (1994). Microbiology.

[18] Bowen WH (2018). Trends Microbiol.

[19] Cavedon K (1993). Oral Microbiology and Immunology.

[20] Lee SF (1996). Infection and Immunity.

[21] Armitage GC (2004). Periodontology 2000.

[22] Eley BM (1999). British dental journal.

[23] De Oliveira JS (2016). Int J Dent.

[24] Cowan MM (1999). Clinical microbiology reviews.

[25] Bast F (2014). The Scientific World Journal.

[26] Cohen MM (2014). Journal of Ayurveda and Integrative Medicine.

[27] Pattanayak P (2010). Pharmacogn Rev.

[28] Jamshidi N (2017). Evid Based Complement Alternat Med.

[29] Gupta D (2014). Journal of Ayurveda and Integrative Medicine.

[30] Agarwal P (2010). Indian J Dent Res.

[32] Ozaslan M (2018). Pak J Biol Sci.

[33] Jayanti I (2018). The Journal of Contemporary Dental Practice.

[34] Ahirwar P (2018). J Indian SocPedodPrev Dent.

[35] Mallikarjun S (2016). Journal of Indian Society of Periodontology.

[36] Kamyab AA (2013). Inflamm Allergy Drug Targets.

[37] Kalita C (2019). J Conserv Dent.

[38] Singh S (2007). Indian J Exp Biol.

[39] Ramamurthy J (2020). Bioinformation.

